# Modularized iCBT‑I self-learn training for university staff—prevention and early intervention in the SARS-CoV-2 crisis: A pilot study

**DOI:** 10.1007/s11818-021-00301-z

**Published:** 2021-02-24

**Authors:** Angelika A. Schlarb, Jan Philipp Fründ, Tatjana Kovacevic, Jasmin Faber

**Affiliations:** Department of Psychology and Sports Science, Bielefeld University, Universitätsstraße 25, 33615 Bielefeld, Germany

**Keywords:** Cognitive behavioral therapy for insomnia, Insomnia/sleep disorder, Video training, Self-lern training, COVID‑19, Kognitive Verhaltenstherapie der Insomnie, Insomnie/Schlafstörung, Videotraining, Selbstlerntraining, COVID‑19

## Abstract

**Background:**

Insomnia is a widespread disease in adults and has a high prevalence rate. As sleep disturbances are a risk factor concerning mental and physical health, prevention and early intervention are necessary. Thus, the aim of this study was to implement a self-learning prevention and early intervention training for university staff members. We adapted an established cognitive behavioral therapy for insomnia (CBT-I) intervention as an online version for use during the severe acute respiratory syndrome coronavirus 2 (SARS-CoV-2) crisis.

**Methods:**

Development and adaptation procedure of the internet-based CBT‑I (iCBT-I) prevention and early intervention training is described. Sessions and topics are shown in detail. The Online Sleep Prevention and Treatment Acceptance questionnaire (OSTA) and the Online Sleep Prevention and Treatment Feedback questionnaire (OSTF) were used to assess acceptance. Sleep problems of university staff members were assessed using the Pittsburgh Sleep Questionnaire (PSQI).

**Results:**

The online-adapted version consisted of seven modules. Contents of sessions and topics were implemented based on video clips. Drawings were added to information regarding sleep and sleep hygiene as well as addressing stress and cognitions. In all, 15 individuals participated in this pilot study. The new iCBT‑I self-learning prevention training was well accepted. In addition, participants scored the online version as helpful based on the OSTA. Prior to online training, 89% of the participants reported impaired sleep quality or insomnia symptoms, and 56% had a PSQI score over 10. After training 78% of participants showed reduced sleep problems according to PSQI and 56% reached clinically significant enhancement. In addition, after training 44% were healthy sleepers.

**Discussion:**

This is the first iCBT‑I prevention and early intervention training for university staff members. The training by participants was very well accepted and they scored the videos as very helpful. Sleep problems decreased after online training. However, further studies with larger samples and more sleep-related assessment strategies, e.g., actigraphy and sleep log, are necessary.

## Abstract (de)

**Hintergrund:**

Insomnie ist eine bei Erwachsenen weit verbreitete Erkrankung und weist eine hohe Prävalenzrate auf. Da Schlafstörungen eine Risikofaktor in Bezug auf die psychische und physische Gesundheit darstellen, sind Prävention und Frühintervention notwendig. Daher ist es das Ziel der vorliegenden Studie, ein Selbstlerntraining zur Prävention und Frühintervention für Hochschulangehörige einzuführen. Aufgrund der SARS-CoV-2-Krise („severe acute respiratory syndrome coronavirus 2“) adaptierten die Autor(inn)en eine etablierte Intervention der kognitiven Verhaltenstherapie bei Insomnie („internet-based cognitive behavioral therapy for insomnia“, CBT-I) als Onlineversion.

**Methoden:**

Die Entwicklung und das Adaptationsverfahren des internetbasierten CBT-I(iCBT-I)-Präventions- und -Frühinterventionstrainings werden beschrieben. Detailliert werden die Sitzungen und Themen dargestellt. Zur Beurteilung der Akzeptanz wurden die Fragebögen Online Sleep Prevention and Treatment Acceptance Questionnaire (OSTA) und Online Sleep Prevention and Treatment Feedback Questionnaire (OSTF) eingesetzt. Mithilfe des Pittsburgh Sleep Questionnaire (PSQI) wurden die Schlafprobleme der Hochschulangehörigen erfasst.

**Ergebnisse:**

Die für den Onlineeinsatz adaptierte Version bestand aus 7 Modulen. Mit Videoclips wurden die Inhalte der Sitzungen und die Gesprächsthemen vorgestellt. Zeichnungen ergänzten Informationen zu Schlaf und Schlafhygiene sowie das Ansprechen von Stress und Kognitionen. Insgesamt gab es 15 Teilnehmer an dieser Pilotstudie. Das neue iCBT-I-Selbstlern-Präventionstraining wurde gut angenommen. Darüber hinaus bewerteten die Teilnehmer anhand des OSTA die Onlineversion als hilfreich. Vor dem Onlinetraining berichteten 89% der Teilnehmer von einer beeinträchtigten Schlafqualität oder von Insomniesymptomen, und 56% wiesen einen PSQI-Wert über 10 auf. Nach dem Training zeigten 78% der Teilnehmer weniger Schlafprobleme gemäß PSQI, und 56% erzielten eine klinisch signifikante Verbesserung. Zudem wiesen 44% nach dem Training einen gesunden Schlaf auf.

**Schlussfolgerung:**

Es handelt sich hier um das erste iCBT-I-Präventions- und Frühinterventionstraining für Hochschulangehörige. Das Training wurde von den Teilnehmern sehr gut angenommen, und sie bewerteten die Videos als sehr hilfreich. Die Schlafprobleme verringerten sich nach dem Onlinetraining. Jedoch sind weitere Studien mit größerer Stichprobe und mehreren Ansätzen zur Schlafbeurteilung, z. B. Aktigraphie und Schlaftagebuch, erforderlich.

## Background

About 20% of adults report poor sleep quality and one third of the German adult population suffer from problems falling asleep or sleeping through the night. Furthermore, about 5.7% of adults met the criteria for insomnia [16]. As various studies demonstrated, sleep complaints are among the most prevalent health concerns, especially among individuals who work, and may lead to adverse consequences as decreases in physical, social and work performance. Apart from health-related consequences the economic costs related to insomnia are also very high because productivity decreases, work-related accidents occur more often and absence times also arise in adults suffering from impaired sleep quality [5]. Prevention and intervention programs addressing sleep quality usually include sleep hygiene and stimulus control components as well as stress management and relaxation techniques [12]. CBT‑I is the state of the art for intervention concerning insomnia showing significant results concerning insomnia symptoms [13]. Current research points toward new technologies showing that treatment for insomnia conducted in a digital way via computer or the internet is also effective [4, 8, 23, 25]. However, studies addressing insomnia in university staff—not university students—are rare. In a 2019 study conducted in Iran, differences of sleep quality in nurses compared to university employees were investigated [15]. Even though the nurses’ sleep quality was significantly worse, 79.6% of the university employees (who were medical hospital staff) suffered from poor sleep quality measured by PSQI [15]. As shown by Mark and Smith, university staff is affected by stress and job-related stress, and report, among other symptoms, sleep problems [10]. Especially work-related rumination was detected to be an influencing factor concerning sleep problems in a working population [2]. Therefore, they summarized that interventions should include components to reduce the work-related cognitive activity which might be important when designing recovery interventions for workers.

Furthermore, since the severe acute respiratory syndrome coronavirus 2 (SARS-CoV-2) outbreak in 2019/2020, various lockdown phases were implemented in order to reduce disease spread. However, besides the virus with its effects, side effects also have to be kept in mind with sleep problems, heightened stress, anxiety and other outcomes [9]. The European CBT‑I Academy formulated practical sleep-related recommendations to support health care services [3]. As telehealth interventions represent a new way to reduce barriers and problems of delivery regarding adequate treatments for sleep problems [23], an online self-learning program seems to be one of the most acceptable and easy to access routes during the pandemic. Only three randomized controlled trials have previously evaluated the efficacy of self-help iCBT‑I in a general working population. To our knowledge, no iCBT-I-based study addressing (a) university staff and (b) based on prevention and early intervention is available. Therefore, the objective of the current study was to develop, implement and examine the acceptance, adherence and efficacy of an iCBT‑I prevention and early intervention self-learning training for sleep problems in university staff. Based on previous studies addressing insomnia in adults [21], we wanted to adapt the structured CBT‑I manual to an iCBT‑I prevention and early treatment training. Adaptation procedure, construction of modules with their contents, and videos and exercises will be described precisely, and acceptance as well as first effects will be evaluated in this pilot study.

We hypothesized that the prevention and early intervention self-learning training (1) would be well accepted by participants, (2) that the video elements would be helpful, (3) technical support was adequate, (4) the themes and module character would be well accepted, (5) exercises would be understandable and helpful and (6) the training would improve the sleep problems and mental health burdens.

## Methods

### Patients and methods

#### Procedure

Before participation, staff members were informed concerning the sleep training via email from the university health service. In addition, interested participants were invited to participate in a kick-off meeting and were sent an information video about the sleep self-learn training. Exclusion criteria were somatic/medication reason for sleep problems. Prior to participation of the training each individual gave their written informed consent. It was advised to finish the self-learn training modules within 7 weeks. Participants had to complete one module before starting the next module (modules described in detailed in Table 1). The modules were structured into four topics—sleep, stress, mindfulness and well-being. Within each of the topics there was a set order of the modules. After each module, participants were asked to give feedback for the module based on the evaluation questionnaires. After 6 weeks participants were reminded to finish the modules and in week 7 an online postinterview was assessed.

**Table 1:** Contents of the prevention training

Module	Contents	Video duration (min)
Module Sleep A	Video 1: Knowledge regarding healthy sleep: sleep stages, sleep–wake regulation, why we sleep, consequences of impaired sleep, unchangeable influencing factors	8:17
	Interactive part 1: Chronotype questionnaire	
	Video 2: Relaxation and sleep	4:47
	Interactive part 2: Relaxation exercise	
Module Sleep B	Video 1: Influencing factors and sleep disorders: changeable influencing factors, sleep disorders, development of a sleep disorder	6:46
	Interactive part 1: Quiz about healthy sleep	
	Video 2: Rules for good sleep: healthy sleep hygiene, healthy sleep environment	6:27
	Interactive part 2: Relaxation exercise	
Module Sleep C	Video 1: Cognitions, rumination and sleep: cognitions as influencing factor, typical sleep disturbing cognitions, contents of sleep disturbing cognitions, sleep myths	7:45
	Interactive part 1: Quiz about sleep myths	
	Video 2: Techniques for nightly ruminations: rumination chair, cognitive designing, how to create healthy sleep-related cognitions	6:31
	Interactive part 2: Exercise for nightly worries and rumination	
Module Stress A	Video 1: Development of stress, stress model, positive sides of stress, physical changes in stress, daily stress, consequences of chronic stress, stress-related behavior and thoughts, stress circle	17:11
	Interactive part 1: Stress Symptom Identification	
	Video 2: Identifying stress, stress and body, emotions, cognitions, and behavior	04:10
	Interactive part 2: Exercise self-release techniques	
Module Stress B	Video 1: Ways out of stress, stress management, changing of primary appraisal, changing of secondary appraisal, changing of coping, changing of reappraisal, subjective start of stress, contents of system	11:00
	Interactive part 1: Day-to-day transfer—how to end stress	
	Video 2: Stress-related cognitions, resources, goals	10:37
	Interactive part 2: Exercise problem solution training	
Module mindfulness	Video 1: What is mindfulness, ways to mindfulness, relation of mindfulness, stress and sleep	11:12
	Interactive part 1: Short mindfulness exercise	
	Interactive part 2: Body scan exercise	
Module well-being	Video 1: Subjective well-being, perspectives and well-being, why is happiness so temporary, ways to more happiness	04:55
	Interactive part 1: Proven vs. ineffective ways to well-being	
	Video 2: Flow, gratitude, social contact, connections between well-being and stress and mindfulness and sleep	08:19
	Interactive part 2: Exercise gratitude diary	

#### Participants

Addressed were university staff members reporting sleep problems or suffering from impaired sleep quality. A total of 15 adults participated in this pilot study; all of them were female. Twelve participants completed pretests and 9 completed pre- and posttests. The age of the participants ranged from 25–63 years, with a mean of 42 years. Daytime impairments were reported by all of the participants. Most participants reported significant sleep problems (8 had PSQI score above 5, one person a PSQI score of 5).

The training was held as online self-learning training without personal or phone contact during training. Diagnostic implementation, construction of prevention training modules and exercises were conducted according to standard ethical guidelines as defined by the Declaration of Helsinki; the study was approved by the ethics committee of Bielefeld University.

### Measurements

#### Pittsburgh Sleep Quality Index (PSQI)

The PSQI was used to assess the subjective sleep quality retrospectively over the past 4 weeks. It consists of 19 items, which are used for a quantitative analysis. Patients’ responses are allocated to a global score, which can range between 0 and 21, and 7 subscales: sleep quality, sleep latency, sleep duration, habitual sleep efficiency, sleep disturbances, use of sleep medication, and daytime dysfunction. Global scores below six represent good sleepers, between six and ten impaired sleepers, and above ten chronic sleep disorders. Based on the reliability coefficients of 0.77 to 0.83 and high specificity and sensitivity in identifying patients with sleep disorders (>80%), the PSQI can be classified as a valid and reliable instrument [3, 6, 11].

#### Online Sleep Treatment Acceptance Questionnaire (OSTA)

To evaluate the acceptance of all contents of the online training, we constructed a specific questionnaire following Schlarb and Brandhorst, [20]. The questionnaire was constructed with regard to the following areas: first, to assess the acceptance of the contents of the treatment and technical issues; second, to provide an evaluation of the training; finally, they had space for remarks and recommendations concerning the training. The questionnaire contained 8 questions; seven questions were based on Schlarb and Brandhorst (2012). Participants rated on a five-point Likert scale (1 = completely applies to me, 2 = rather applies to me, 3 = I don’t know, 4 = rather doesn’t apply to me, 5 = doesn’t apply to me at all).

#### Online Sleep Treatment Feedback Questionnaire (OSTF)

In addition to the above-mentioned OSTA questionnaire, participants were also asked to fill in the OSTF. This questionnaire contained 2 questions for every video and interactive element of the program, regarding the technical aspects and usefulness of the online training. Participants rated the items on a five-point Likert scale (1 = very much, 2 = much, 3 = moderate, 4 = little, 5 = none).

#### Online Sleep Treatment Evaluation Questionnaire (OSTE)

After the program, participants were asked to fill in the OSTE. This questionnaire contained 6 questions regarding the overall evaluation of the online training. Participants rated the items on a five-point Likert scale (Depending on the question: very good/very much = 1 good/much = 2 moderate/a little = 3 bad/little = 4 very bad/nothing = 5).

#### Feedback interview

After training, an online interview was implemented (1 h) to assess further qualitative data concerning structure, time, contact possibilities and other aspects beyond sleep parameters. One part of the interview was structured, whereas the second part was not structured. Two of the authors independently wrote a protocol.

### Online prevention and early intervention adaptation (iCBT-I prevention for adults)

The original CBT‑I program was created for chronic insomnia disorder and nightmare disorder based on ICD-10, DSM‑5 or ICSD‑3 criteria [17, 19]. Contents of the CBT‑I were formalized in a manual [17] and effects for insomnia population also with comorbid depression was published [18, 21]. For a more detailed description of the treatment components see Schlarb (in press) [17]. Adaptation of the sessions was relatively easy as the original manual gives detailed advice concerning behavior strategies coping with the sleep problems and various elements based on imaginary techniques or clinical hypnotherapy. However, all strategies had to be adapted for online prevention and early intervention modality (advice, exercises, etc.). In addition, the drawings were added to PowerPoint (Microsoft) slides to enhance readability and understanding of sleep-related information and training instructions. Furthermore, video clips were also created to enhance the information sections and explain for example the relaxation strategies. All exercises for at home were provided in written form and explained in detail. For more detailed information regarding the adapted prevention and early intervention module system see Table 1.

### Analysis

Various data were assessed. Besides acceptance, feasibility and adherence of online prevention training, data analysis was conducted qualitatively and quantitatively. In detail, each of the modules was evaluated. In addition, the training as a whole was also evaluated. Besides the qualitative data, quantitative and qualitative data of 12 adults regarding the modules of the training and sleep and emotional health were assessed. The significance level was set at *p* = 0.05. Effect sizes are reported according to Cohen’s d.

#### Qualitative analysis

Through this method we aimed to investigate the experiences and opinions of the participants about the program on a deeper level, in order to evaluate and improve the program in the long run. The foundation of our qualitative analysis were the open answers of the structured questions and the protocol of the feedback interview. For the analysis we choose the method of qualitative evaluation based on work by Kuckartz et al. [7] for efficient evaluation while using systematic qualitative methodology. First, we specified our aims based on our overall hypothesis and explored the qualitative data. The authors presented suggestions for categorization, which were discussed, combined and refined in discussions. The category system is presented in Table 2.

**Table 2:** Categories for qualitative evaluation

Course Content	*Content Aspects (CA)*—content aspects related to information transfer (what content, quantity, etc.)
	*Exercises (EX)*—content aspects related to the exercises
Implementation of the Course	*Course Procedure and Structure (PS)*—structure of the program, freedom of choice of the module blocks, sequence of the modules, structure and length of the modules
	*Technical Implementation of the Course (TI)*—this includes everything that roughly falls into the technical handling
	*Feedback on the Course Material (CM)*—aspects of the material: presentation, videos, speech, download material
Effects on participants	*Effects on Participants (EP)*—what do the participants say the course has changed for them? Experiences with the program, etc.
Other comments	*Other Comments (OC)*—feedback related to, for example, questionnaires or other aspects

The answers of the participants were independently rated by TK and JF, with AS deciding cases that were not unanimous. The rated data were organized into positive feedback and suggestions for improvement/negative feedback and reported via frequency table and evaluation report.

## Results

From the 12 participants participating in the iCBT‑I prevention self-learning training, feedback data regarding online training is available, whereas post-measurements of 9 participants were assessed. In addition, participants returned the sleep-related questionnaire (PSQI) and other questionnaires as mental health-related information (WHO-5).

One of the main objectives was to evaluate the acceptance of online iCBT‑I prevention training from the participants’ view. Therefore, in the following sections, we first describe the results of the OSTA and the OSTF regarding acceptance of the prevention training but also results of qualitative research.

For outcomes concerning sleep and mental health we present pre- and postmeasurement data of participants who returned the postmeasurement questionnaires (*n* = 9).

### Acceptance

To measure acceptance, participants rated all topics of the online self-learn training with the OSTA on a five-point Likert scale. In sum, all participants rated the information given in the modules as helpful. In all, 33% rated the material as very helpful. In addition, we received 24 module-feedback questionnaires (Tables 3 and 4).

**Table 3:** Questions of Online Sleep Prevention and Treatment Acceptance questionnaire (OSTA) over all modules

Questions OSTA	M (SD)
The theoretical and practical knowledge was comprehensibly taught	1.50 (0.75)
The topic appealed to me	1.62 (0.82)
I will implement strategies of the module	1.69 (0.52)
The information of the module was helpful to me	1.81 (0.78)
There were enough training possibilities	1.82 (0.85)
I got new impulses for my everyday life	2.13 (1.00)
Contents of the module were new for me	2.96 (1.26)

Scores ranged from 1 (completely applies to me) to 5 (doesn’t apply to me at all)

*M* mean, *SD* standard deviation

**Table 4:** Means of all modules based on Online Sleep Prevention and Treatment Acceptance questionnaire (OSTA) questionnaires

Questions OSTA	M (SD)	M (SD)	M (SD)	M (SD)	M (SD)	M (SD)	M (SD)
Module	Sleep A	Sleep B	Sleep C	Stress A	Stress B	Mindfulness	Well-being
The theoretical and practical knowledge was comprehensibly taught	1.67 (1.11)	1.50 (0.71)	1.20 (0.40)	2.00 (0.71)	1.25 (0.43)	1.25 (0.43)	1.67 (0.47)
The topic appealed to me	1.50 (0.50)	1.75 (0.97)	1.20 (0.40)	2.00 (0.71)	1.25 (0.43)	2.00 (1.22)	1.67 (0.47)
I will implement strategies of the module	1.83 (0.37)	1.75 (0.43)	1.60 (0.49)	2.25 (0.43)	1.25 (0.43)	1.50 (0.50)	1.67 (0.47)
The information of the module was helpful to me	2.50 (1.12)	2.00 (0.00)	1.20 (0.40)	2.00 (0.71)	1.25 (0.43)	1.75 (0.83)	2.00 (0.00)
There were enough training possibilities	2.00 (1.00)	1.50 (0.50)	1.60 (0.49)	2.25 (1.09)	1.25 (0.43)	1.50 (0.50)	2.67 (0.94)
I got new impulses for my everyday life	2.50 (1.12)	2.50 (0.87)	1.40 (0.49)	2.75 (0.83)	1.25 (0.43)	2.50 (1.12)	2.00 (0.00)
Contents of the module were new for me	3.17 (1.21)	3.50 (0.87)	2.20 (0.98)	3.50 (0.87)	2.00 (1.22)	3.00 (1.58)	3.33 (0.94)

Scores ranged from 1 (completely applies to me) to 5 (doesn’t apply to me at all)

*M* mean, *SD* standard deviation

In addition to the contents of the training, we also evaluated the implemented videos in detail. Most of the participants liked the videos. Detailed results regarding the OSTF are presented in Table 5.

**Table 5:** Online Sleep Treatment Feedback Questionnaire (OSTF). “How useful was [part of program] for you?”

Module	Mean (SD)	Feedback frequency
Sleep A	3.12 (0.60)	6
Sleep B	2.63 (0.58)	8
Sleep C	2.13 (0.92)	5
Stress A	2.50 (0.90)	4
Stress B	1.75 (0.97)	4
Mindfulness	2.25 (0.50)	4
Well-being	2.33 (0.50)	3

Answer Format: 1 = very much, 2 = much, 3 = moderate, 4 = little, 5 = none

*SD* standard deviation

Overall evaluation of the training was conducted with OSTE. In Table 6 the results are presented in detail.

**Table 6:** Online Sleep Treatment Feedback Questionnaire (OSTF)

OSTE questions	Mean (SD)
How would you rate the usability of the program?	1.67 (0.71)
How did you like the program overall?	2.22 (0.44)
Were you able to learn anything through the program?	2.89 (0.60)
Did you change anything about your behavior in general because of the program?	3.22 (0.44)
Do you think the program had a positive impact on you?	2.67 (0.50)
How much benefit did the program have for you overall?	2.89 (0.60)

Answer formats varied: very good/very much = 1, good/much = 2, moderate/a little = 3, bad/little = 4, very bad/nothing = 5

### Qualitative feedback (open answer format)

#### Course content

##### Content aspects (CA).

Regarding information transfer, the participants rated the program as understandable and remarked positively that the information directly addressed the matter and the information parts were appropriate in length. Although some of the content was already known, there were parts that participants reported as being of special interest. Beside factors like everyday mindfulness, flow and individual differences in sleep behavior, information on factors influencing sleep, “*there are things you can’t change regarding sleep […] That is reassuring. But there are also some things you can change.*” was highlighted multiple times as being very valuable.

##### Exercises (EX).

In the answers to the evaluations questionnaires there was a lot of positive feedback regarding the exercises. Nine comments were given about how great the various exercises were, “*Breathing exercise is great, just like a prescription*”. However, there were some remarks about the wording and illustration that could be improved for better clarity. The integratability into day-to-day life was praised by five participants, “*The breathing exercise is easy to integrate into everyday life*”, as well as the implementability, “*Easy to implement*”.

#### Implementation of the course

##### Technical implementation of the course (TI).

Concerning the technical implementation there were positive comments, especially mentioned were the LernraumPlus interface and the general structure of the program. A suggestion for improvement was to use more variety in the form the content is presented. Particularly more audio options would be appreciated, since these would offer more flexibility to use the program in daily life and get away from the screen. It was remarked that the used online tools need practice and that one has to get used to using them, but that they also work quite well and hold certain benefits.

##### Course procedure and structure (PS).

The overall structure of the program was received very well. Five comments emphasized that the procedure of having certain main topics, which order is chosen freely is a good concept, *“Very good that you can swap the order between main topics”*. Some participants suggested to offer additional individual consulting appointments to deal with individual questions and topics, if that would be feasible.

##### Feedback on the course material (CM).

This category got most feedback in comparison with the others (33 comments were in the category CM). Eight comments stated that the drawings were pretty and descriptive: *“the small drawings are pretty and visually emphasize what is being said”. *The way of speaking yielded mixed reactions: five comments liked the calm style that was used (*“I could follow the video very well, it was pleasantly spoken, it took the pressure out overall”*) but two comments did not see that equally well (*“With the speaking text, I would like to see a more positive mood conveyed”*). The background noise, which occurred in some recordings, was disliked especially during the exercise: *“[…] background noise on the recording distracted me and it was not very soothingly spoken.” *The visibility of the trainers in the course videos was also received variably. While three comments rated this as a nice and relatable feature, in two comments it was found distracting: *“With the Panopto video, I would have liked to have only the audio and not have the speaker in the picture. That was distracting to me”. *One comment stated that differing speakers helped to maintain attention throughout the course.

#### Effects on participants (EP)

There were some quite encouraging effects as experiencing better sleep afterwards. Not all parts were helpful though, exercises were tried but did not have an effect: *“I already use such mindfulness exercises, but I doubt that they help me. I use them at night when I can’t sleep. Often they do not help”.* Sleep was noticeably improved through the awareness that sleep interruptions in the night do not have to be a big issue to worry about because worrying itself is a problem.

#### Other comments (OC)

Participants described that they had various differing sleeping conditions which were not completely included in the questionnaires. This included sharing of bed with snoring partners half of the weekly nights and changing apartments on the weekend: “*My sleeping situation is pretty much as stated in the questionnaire (alone) half the time, the other half of the time I sleep in a bed with my partner.*”. Mindfulness was described as a difficult concept to work with in two comments because of exaggerated claims which were encountered in contexts other than those in the program. An especially reflective handling of the term was suggested. Three comments stated that they would have liked further integration with other resources, e.g., courses, online literature or links (Table 7).

**Table 7:** Overview of qualitative feedback

	Freq.	Cat.
*Positive feedback*		
The different exercises (54321, breathing exercise, self-release, body scan, if–then exercise) were very well received	9	EX
The drawings are pretty and descriptive	8	CM
The exercises are easy to implement, can be easily integrated into everyday life	5	EX
The system where you can switch the order between main topics works well	5	PS
Concerning the way of speaking, there was positive feedback	5	CM
The awareness of stress sensations was improved and that was very helpful	4	EP
The contents of the program were understandable and to the point	3	CA
Information on factors influencing sleep was good	3	CA
Everyday mindfulness, flow and individual sleep differences highlighted was of particular interest	3	CA
The online tools require practice/getting used to, but they work and also bring benefits	3	TI
The visibility of the speakers in the video was nice	3	CM
The technical implementation is well done	2	TI
The awareness that poor sleep or interruption of sleep is not a big issue helps improve sleep	2	EP
It is good to have various speakers for better attention throughout the program	1	CM
Concrete action was triggered by the program	1	EP
*Suggestions for improvement/negative feedback*		
Speech rate of the videos was too slow (especially for sleeping parts)	7	CM
Background noise was unpleasant, especially during the exercises. Good audio quality is important	4	CM
Some contents were already known	3	CA
The exercises are sometimes formulated somewhat unclearly and should be better illustrated	3	EX
More variety in the form of presentation would be good, especially more audio options would be appreciated	3	TI
Not everything is helpful	3	EP
Additions to the prequestionnaire, not all topics considered relevant were asked about	3	OC
Extension of the offer by individual consulting appointments desired if feasible	3	PS
Concerning the way of speaking, there was negative feedback	2	CM
It was distracting to see the speaker in the video	2	CM
The term “mindfulness” is difficult for some people and should be used in a reflective way	2	OC

*Freq.* Frequencies, *Cat.* Categories (*CA* content aspects, *EX* exercises, *TI* technical implementation of the course, *PS* course procedure and structure, *CM* feedback on the course material, *EP* effects on participants, *OC* other comments)

### First effects

Besides feasibility, acceptance and adherence we also evaluated the sleep-related effect in this pilot study. Therefore, sleep-related feedback was assessed by PSQI. In addition, mental health will be reported, pre–post data will be presented.

**Fig. 1:**
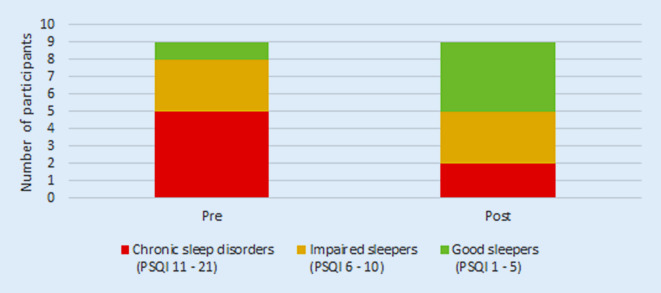
Changes of Pittsburgh Sleep Quality Index (PSQI) scores prior to and after training

Overall, 56% of the participants slept better after the training according to self-report with a significant clinical change (Fig. 1). On average, participants experienced better overall sleep quality (PSQI scores) after the program (M = 7.44, SD = 4.13), than before the program (M = 10.33, SD = 3.97). This difference, −2.89, BCa 95% CI [−4.55, −1.22], was significant t(8) = −3.0, *p* = 0.017, and represented a large effect, d = 2.89.

On average, participants slept longer after the training (M = 0.67, SD = 1.11), than before the program (M = 1.67, SD = 0.87). This difference, −1.0, BCa 95% CI [−1.67, −0.33], was significant t(8) = 2.68, *p* = 0.028, and represented a large effect, d = 1.12.

On average, participants experienced no change in sleep quality due to bad dreams (“had bad dreams”): after the program (M = 1.67, SD = 1) was the same as before the program (M = 1.67, SD = 1). However, the change −0.00, BCa 95% CI [−0.33, 0.33], was not significant (t(8) = 0, *p* = 1.).

#### Insomnia symptoms

Prior to training, 56% participants reported insomnia symptoms as prolonged sleep onset latency of more than 30 min, or nocturnal wakenings, and daytime impairment as tiredness.

Insomia symptoms were reduced as participants fell asleep faster afterwards (M = 1.56, SD = 0.73) than before training (M = 2.0, SD = 1.0). The enhancement, −0.44, BCa 95% CI [−0.78, −0.11], was significant t(8) = −2.53, *p* = 0.035, with a medium-sized effect, d = 0.53. Furthermore, insomnia symptoms as prolonged sleep onset latency (more than 30 min) was reduced after training (M = 2.44, SD = 0.73) than before the program (M = 3.11, SD = 0.93). This difference, −0.67, BCa 95% CI [−1.11, 0.22], was also significant t(8) = 2.82, *p* = 0.022, with a medium effect size, d = 0.71. In addition, problems sleeping through the night also improved after the program (M = 3.11, SD = 1.27), in contrast to before the program (M = 3.33, SD = 1.11). However, this difference, −0.22, BCa 95% CI [−0.89, 0.44], failed to reach significance t(8) = −0.61, *p* = 0.56. In addition, daytime tiredness decreased from M = 1.44 (SD = 0.73) to PM = 1.22 (SD = 0.67) after training. Furthermore, participants reported improved subjective sleep quality (PSQI scores) after training (M = 1.22, SD = 0.44) than before (M = 1.89, SD = 0.78). This difference, −0.67, BCa 95% CI [−0.89, −0.33], was significant t(8) = −4.00, *p* = 0.004, with a medium-sized effect, d = 0.5.

The mental health score (WHO‑5 score) was M = 9.89 (SD = 4.01) prior to online training and M = 10.33 (SD = 3.36) after training. Values less than 13 may be an indicator for depression. A higher value indicates greater well-being (maximum 25).

## Discussion

The main goal of this study was to develop an online prevention and early intervention training for university staff members mostly reporting impaired sleep quality or insomnia symptoms. We wanted to assess acceptance and feasibility and to evaluate sleep-related outcomes in this pilot study. As the SARS-CoV‑2 crisis is associated with more stress and reduced sleep quality [9], new ways regarding sleep help were required instead of face-to-face training. Therefore, we constructed an online prevention version of an established CBT‑I based training.

### Acceptance and feasibility

Overall, the adapted version of the training was well accepted. Most scored the videos as helpful and entertaining. However, some suggested the possibility of adding live sessions with trainers. We wanted to include a participative development perspective by combining open feedback and structured evaluation in order to obtain a detailed view of participants’ subjective estimations and preferences. Through this method, we received various hints to improve the program, for example, the option to individualize aspects of the program. In online intervention formats, adherence is an important factor, thus, enhancing motivation is important; one way to achieve this is to offer individualization. Participatory approaches for developing and adapting online programs can profit from qualitative data because formerly unidentified factors can be included.

### Sleep-related outcomes

Most participants rated the online prevention training as helpful to enhance their sleep quality and improve sleep-related problems. These results are in line with other online interventions. For example, in 2020 Behrendt et al. [2] addressed a sample of 177 workers who were randomized to receive either iCBT‑I (*n* = 88) or controls (*n* = 89). Their intervention was also a web-based training consisting of six 1‑week modules. In line with our study, the training was self-help oriented. However, their participants also received technical support. In their study iCBT‑I participants reported significantly lower insomnia severity scores at the postintervention assessment which is also the case in our pilot study. Furthermore, the findings are in line with the review of Spiegelhalder et al. [23], reporting also high acceptance rates for iCBT‑I. However, this is the first online self-learning training addressing university staff. But we have to keep in mind that the mean PSQI scores were still above the cut-off directly after training but clinically lower. Most participants reached clinically significant changes. Furthermore, insomnia symptoms decreased—especially sleep onset latency and sleep quality which is also reported by face-to-face treatment effects [21]. According to the participants’ feedback, the theoretical and practical knowledge was comprehensible. Furthermore, the topic appealed to them. In addition, they reported implementing the strategies in their daily routine.

According to the feedback questionnaire, the materials were helpful to change their sleep problems. In addition, all rated the videos helpful. This is in line with previous iCBT‑I studies [14, 23, 24]. However, our findings widen the results pointing towards prevention training.

### Rumination, mental health and other outcomes

Concerning other mental health outcomes our pilot study shows that besides sleep-related enhancements, mental health was also slightly better after training resulting in reduced scores concerning depression and a higher quality of life. These results are in line with Behrens et al., who reported significant differences for cognitive activity and for mental and work-related health, especially work-related rumination and worry as mediators on sleep [2].

### Application

Concerning the training application, in contrast to a previous study addressing children, our study had no personal contact [22] and no meeting each other in person [20]. However, some of the participants recommended the possibility of additive personal contacts for questions or further information (e.g., concerning specific sleep-related questions of asthma patients). Overall, the study was also well accepted regarding application. As we addressed a specific group (university staff) showing a high prevalence of sleep problems, prevention and early intervention is very important.

### Limitations

There are some limitations of the study which should be mentioned. As only 12 individuals participated in this pilot study addressing the adaptation and feasibility of an online self-learning format, the results have to be considered with caution. However, we received detailed feedback from participants addressing the self-learning format, the videos and drawings and also feedback concerning technical issues.

However, future online prevention studies should implement more detailed sleep measures as extensive sleep logs and also objective measurements as actigraphy. As this study was a pilot study, we had no further follow-up measurements. Future studies implementing long-term outcomes concerning sleep and mental health are required. However, the aim of this study was to adapt the manual-based CBT‑I intervention for adults in an online self-learning prevention and early intervention format. Assessment of acceptance and feasibility of this adapted version by participants suffering from impaired sleep quality or insomnia symptoms was positive.

## Conclusions

As this study shows, online prevention training based on CBT‑I as a self-learn version for university staff members was highly accepted and effective concerning sleep. The SARS-COV‑2 crisis forces new ways of intervention integrating recommendations by the European CBT‑I academy [1]. This pilot study shows that online training is possible for university staff and further that training instructions, exercises and homework instructions can be transferred into video clips and drawings with the help of an electronic slide presentation.
